# Supplementary material for: Plasma cathepsin D as an early indicator of alcohol-related liver disease
Source: JHEP Rep. 2024 May 9;6(9):101117. doi: 10.1016/j.jhepr.2024.101117 (PMC11388167; doi:10.1016/j.jhepr.2024.101117)
Supplement: Multimedia component 1 [file mmc1.pdf]

Plasma cathepsin D as an early indicator of alcohol-related liver disease

Mengying Li, Tom Houben, Albert V. Bitorina, Dennis M. Meesters, Mads Israelsen, Maria Kjærgaard, Ger H. Koek, Tim Hendrikx, Jef Verbeek, Aleksander Krag, Maja Thiele, Ronit Shiri-Sverdlov

Table of contents

Fig. S1……………………………………………………………………………2
Fig. S2……………………………………………………………………………3
Fig. S3……………………………………………………………………………4
Table S1…………………………………………………………………………5
Table S2…………………………………………………………………………7
Table S3…………………………………………………………………………8
Table S4…………………………………………………………………………9
Table S5………………………………………………………………………...10
Table S6………………………………………………………………………...11
Table S7………………………………………………………………………...12
Table S8………………………………………………………………………...13

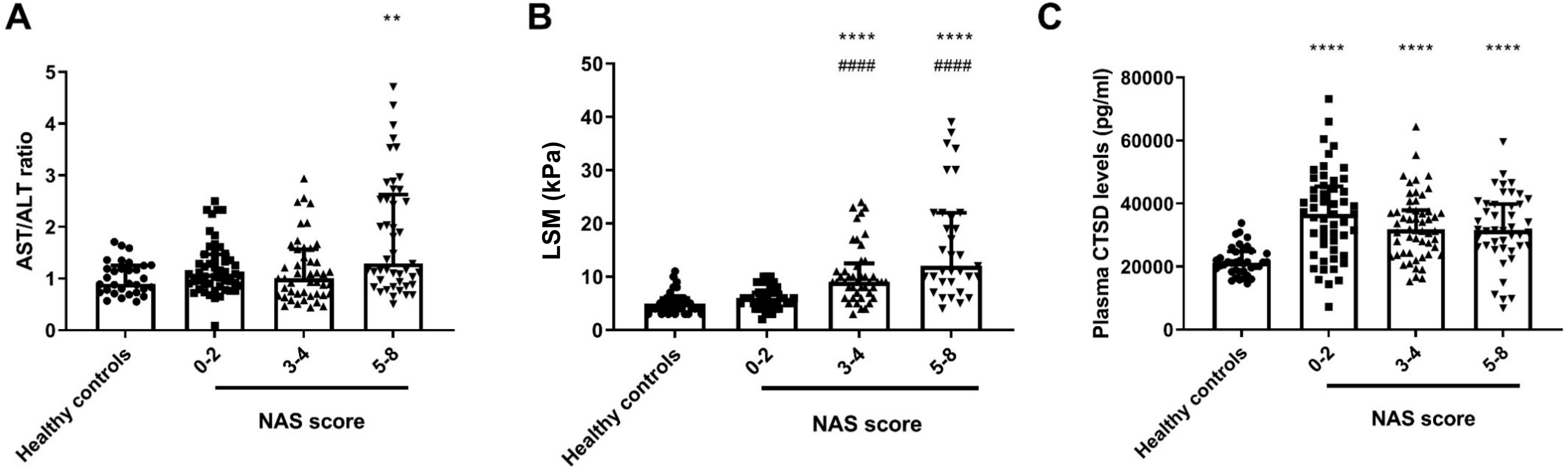

Fig. S1: Plasma CTSD levels and hepatic disease markers increased in MetALD/ALD patients classified according to NAS score

AST/ALT ratio, liver stiffness measurement (LSM) and CTSD levels were analyzed in healthy controls (n=40) and MetALD/ALD patients (n=152) and classified according to the NAFLD NAS-CRN system. The composite NAS score consisted of the sum of steatosis, ballooning and lobular inflammation. ** $p<0.01$, **** $p<0.0001$ compared to healthy controls and #### $p<0.0001$ compared to NAS score 0 (Kruskal-Wallis test). All error bars are median with interquartile range.

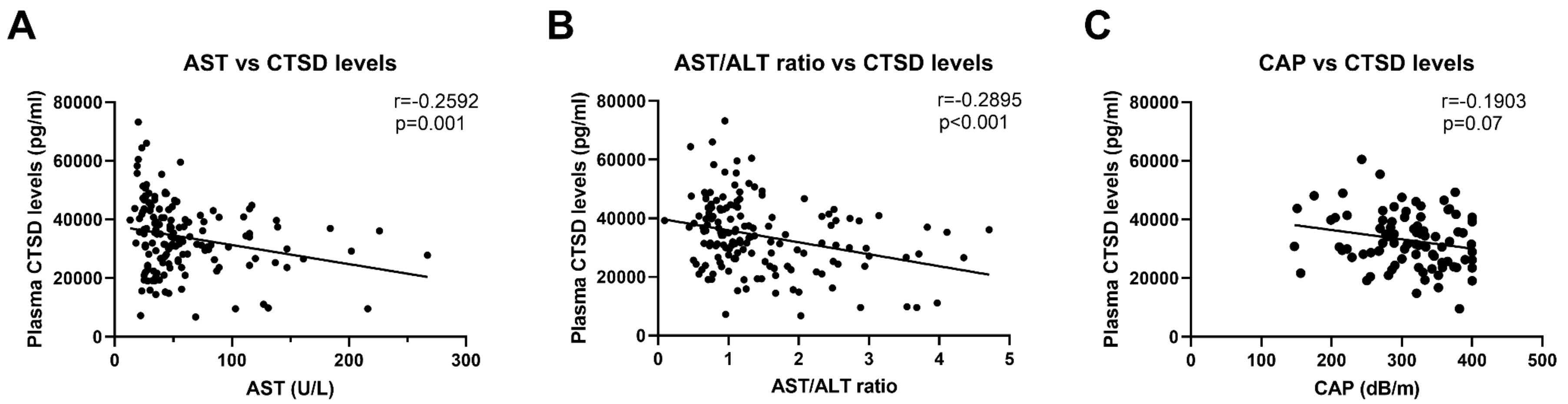

Fig. S2: Plasma CTSD levels correlated with AST and AST/ALT ratio in MetALD/ALD patients.

(A) Plasma CTSD levels vs. AST (n=148), (B) plasma CTSD levels vs. AST/ALT ratio (n=148) and (C) and plasma CTSD levels vs. CAP (n=89). Spearman's correlation was performed. $p<0.05$ was considered statistically significant.

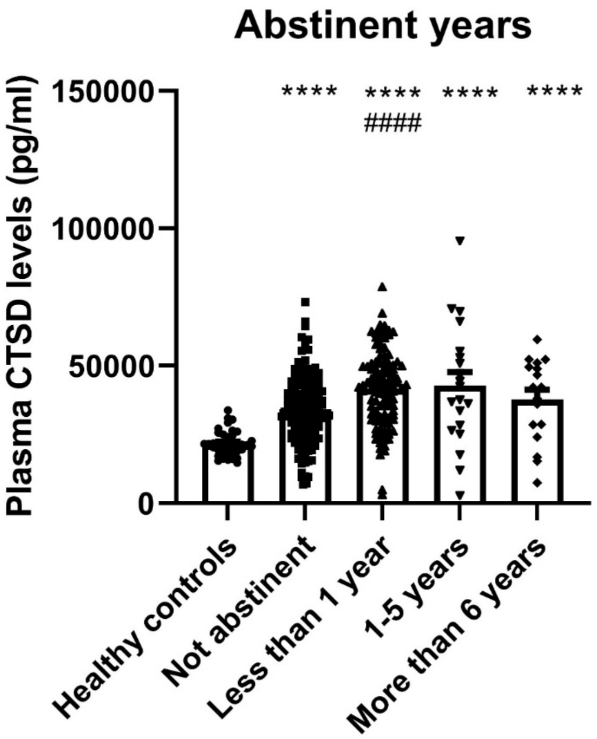

Fig. S3: Plasma CTSD levels during prolonged periods of self-reported abstinence

**** p<0.0001 compared to healthy controls and # # # # p<0.0001 compared to patients that were not abstinent (indicated in graph as 'not abstinent') (Kruskal-Wallis test). All error bars are median with interquartile range. (healthy controls, n=38; not abstinent, n=156; less than 1 year of abstinence, n=112; 1-5 years of abstinence, n=20; more than 6 years of abstinence, n=17)

Table S1: Clinical characteristics of MetALD/ALD patients based on NAS score

Parameters	Healthy controls (n=40)	NAS score 0 (n=20)	NAS score 1-4 (n=86)	NAS score 5-8 (n=45)	p-value (score 0 vs. healthy controls)	p-value (score 0 vs. score 1-4)	p-value (score 0 vs. score 5-8)
**Gender (F/M)**	9/31	6/14	19/67	6/39	0.535	0.458	0.165
**Age (y)**	53±1.6	50±2.8	57±1.1	58±1.4	0.243	0.004*	0.004*
**BMI (kg/m2)**	26±0.5	26±1.0	28±0.7	27±0.8	0.546	0.054	0.186
**HOMA-IR**	2.8±0.4	7.2±5.2	5.9±0.9	7.0±1.1	0.406	0.661	0.939
**Insulin (pmol/L)**	69.4±8.0	109.9±59.0	129.7±17.6	134.0±15.4	0.349	0.669	0.599
**Fasting glucose (mmol/L)**	5.9±0.2	6.4±0.4	7.0±0.2	7.4±0.3	0.194	0.228	0.091
**Hemoglobin A1c (mmol/mol)**	37±1.0	38±2.8	37±1.2	37±1.4	0.918	0.977	0.756
**LDL cholesterol (mmol/L)**	3.3±0.1	2.8±0.2	2.8±0.1	2.6±1.1	0.056	0.989	0.393
**HDL cholesterol (mmol/L)**	1.3±0.08	1.4±0.11	1.5±0.06	1.5±0.11	0.796	0.430	0.306
**Triglyceride (mmol/L)**	1.3±0.1	1.8±0.2	2.2±0.2	1.4±0.1	0.043*	0.482	0.098

**CAP (dB/m)**	270±9	227±25	313±8	314±11	0.070	0.001*	0.001*
**ALT (U/L)**	31±3	26±3	49±5	58±4	0.329	0.040*	0.000*
**AST (U/L)**	28±2	27±2	47±3	91±8	0.895	0.000*	0.000*
**AST/ALT ratio**	1.01±0.06	1.16±0.09	1.30±0.09	1.82±0.17	0.148	0.273	0.001*
**LSM (kPa)**	7.0±1.5	5.4±0.5	13.6±1.6	27.5±3.7	0.471	0.000*	0.000*
**MELD**	6.7±0.1	6.6±0.2	7.2±0.2	8.0±0.3	0.498	0.208	0.002*
**CRP (mg/L)**	3.2±0.5	3.6±1.5	7.4±2.7	10.8±2.4	0.744	0.541	0.014*
**Plasma CTSD levels (pg/mL)**	22858.54±1059.15	37737.03±3403.45	33949.17± 1235.81	32189.32± 1663.63	0.000*	0.215	0.104

Data are represented as mean ± SEM. Data are statistically analysed by performing an independent sample t-test using SPSS software, version 27.0 (SPSS, Chicago, IL, USA). BMI: body mass index; HOMA-IR: homeostasis model of insulin resistance; LDL: low-density lipoprotein; HDL: high-density lipoprotein; CAP: controlled attenuation parameter; ALT: alanine transaminase; AST: aspartate transaminase; LSM: liver stiffness measurement; MELD: model of end stage liver disease; CRP: C-reactive protein. * $p<0.05$ is statistically significant.

Table S2: Plasma CTSD levels independently associated with plasma triglycerides in NAS score 0 MetALD/ALD patients and healthy controls

Dependent variable: plasma CTSD levels

	**LDL cholesterol**			**Triglyceride**		
**Models**	**p value**	**Standardized coefficients β**	**Adjusted R square**	**p value**	**Standardized coefficients β**	**Adjusted R square**
**Model 1**	0.242	-0.156	0.007	0.030*	0.286	0.065
**Model 2**<br>Model1+age	0.668	-0.060	0.051	0.034*	0.270	0.123
**Model 3**<br>Model 2+BMI	0.690	-0.057	0.034	0.026*	0.293	0.116
**Model 4**<br>Model 3+sex	0.698	-0.055	0.025	0.034*	0.285	0.103

Data were analyzed by multiple linear regression: Model 1, simple regression; Model 2, model 1 + adjustment for age; Model 3, model 2 + adjustment for BMI; Model 4, model 3 + adjustment for sex. * p<0.05 is statistically significant.

Table S3: Plasma CTSD levels are associated with hepatic disease markers in MetALD/ALD patients

Dependent variable: plasma CTSD levels

	LSM			**AST/ALT ratio**			**AST**			**ELF**			**GGT**		
**Models**	**p value**	**Standardized coefficients β**	**Adjusted R square**	**p value**	**Standardized coefficients β**	**Adjusted R square**	**p value**	**Standardized coefficients β**	**Adjusted R square**	**p value**	**Standardized coefficients β**	**Adjusted R square**	**p value**	**Standardized coefficients β**	**Adjuste R squar**
**Model 1**	0.005*	-0.231	0.047	0.000*	-0.306	0.087	0.004*	-0.238	0.050	0.045*	-0.163	0.020	0.052	-0.158	0.025
**Model 2** Model1+age	0.007*	-0.224	0.049	0.000*	-0.302	0.086	0.003*	-0.240	0.052	0.072	-0.151	0.015	0.043*	-0.165	0.034
**Model 3** Model 2+BMI	0.007*	-0.224	0.042	0.000*	-0.347	0.093	0.002*	-0.259	0.050	0.070	-0.154	0.009	0.039*	-0.173	0.035
**Model 4** Model 3+sex	0.009*	-0.218	0.038	0.000*	-0.344	0.092	0.002*	-0.259	0.051	0.098	-0.142	0.008	0.045*	-0.168	0.043

Data were analysed by multiple linear regression: Model 1, simple regression; Model 2, model 1 + adjustment for age; Model 3, model 2 + adjustment for BMI; Model 4, model 3 + adjustment for sex. * $p<0.05$ is statistically significant.

Table S4: Overview of the comparison between the different single AUCs

	Standard Error [a]	z statistic	95% Confidence Interval[b]	P-value
CTSD levels vs. AST/ALT ratio	0.0702	2.689	0.0512 - 0.326	P=0.0072
CTSD levels vs. TE	0.0609	0.664	-0.0789 - 0.160	P=0.5070
AST/ALT ratio vs. TE	0.0728	2.037	0.00561 - 0.291	P=0.0416

[a] DeLong et al., 1988

[b] Binomial exact

Table S5: Different statistical criteria related to the diagnostic value of plasma CTSD, AST/ALT ratio and TE for distinguishing MetALD/ALD from healthy controls

	Cut-off value	Sensitivity %	Specificity %	PPV %	NPV %	LR+	LR-	Accuracy%
CTSD levels	≥21933.10 (pg/ml)	87	68	92	53	2.7	0.2	83
LSM	≥6.5 (kPa)	69	74	92	34	2.7	0.4	70
AST/ALT ratio	≥1.37	36	90	94	24	3.7	0.7	45

PPV: positive predictive value; NPV: negative predictive value; LR+: positive likelihood ratio; LR-: negative likelihood ratio.

Table S6: Overview of the comparison between the different AUCs

	Standard Error [a]	z statistic	95% Confidence Interval[b]	P-value
AST/ALT ratio vs. CTSD + AST/ALT ratio	0.0527	4.609	0.140 - 0.346	P<0.0001
LSM vs. CTSD + LSM	0.0418	2.229	0.0113 - 0.175	P = 0.0258
AST/ALT ratio + LSM vs. CTSD + AST/ALT ratio + LSM	0.0467	3.717	0.0821 - 0.265	P = 0.0002

[a] DeLong et al., 1988

[b] Binomial exact

Table S7: Different statistical criteria related to the diagnostic value of combining hepatic disease markers for distinguishing MetALD/ALD from healthy controls

	Sensitivity %	Specificity %	PPV %	NPV %	LR+	LR-	Accuracy%
CTSD + LSM + AST/ALT ratio	85	84	96	55	5.3	0.2	85
CTSD + LSM	85	84	96	55	5.3	0.2	85
CTSD + AST/ALT ratio	70	94	98	40	10.8	0.3	74
AST/ALT ratio + LSM	47	94	97	28	7.3	0.6	55

PPV: positive predictive value; NPV: negative predictive value; LR+: positive likelihood ratio; LR-: negative likelihood ratio.

Table S8: Plasma CTSD levels during prolonged periods of self-reported abstinence

Years of abstinence	Mean plasma CTSD levels (pg/ml)	N
Healthy controls	21735.5	38
No abstinent	34100.3	156
Less than 1 year	41688.2	112
1-5 years	42786.3	20
6-10 years	30851.4	7
11-20 years	50079.5	6
21-30 years	43824.3	2
more than 30 years	18074.0	2
Total	37654.7	305

Data are represented as mean
