# Supplementary material for: Plasma cathepsin D as an early indicator of alcohol-related liver disease
Source: JHEP Rep. 2024 May 9;6(9):101117. doi: 10.1016/j.jhepr.2024.101117 (PMC11388167; doi:10.1016/j.jhepr.2024.101117)
Supplement: Multimedia component 4 [file mmc4.pdf]

Plasma cathepsin D as an early indicator of alcohol-related liver disease

Mengying Li[1], Tom Houben[1], Albert V. Bitorina[1], Dennis M. Meesters[1], Mads Israelsen[2], Maria Kjærgaard[2], Ger H. Koek[3], Tim Hendrikx[4], Jef Verbeek[5], Aleksander Krag[2], Maja Thiele[2], Ronit Shiri-Sverdlov[1,*]

JHEP Reports **2024**. vol. 6 | 1–10

Check for updates

Background & Aims: People who drink alcohol excessively are at increased risk of developing metabolic dysfunction and alcohol-related liver disease (MetALD) or the more severe form alcohol-related liver disease (ALD). One of the most significant challenges concerns the early detection of MetALD/ALD. Previously, we have demonstrated that the lysosomal enzyme cathepsin D (CTSD) is an early marker for metabolic dysfunction-associated steatohepatitis (MASH). Here, we hypothesized that plasma CTSD can also serve as an early indicator of MetALD/ALD.

Methods: We included 303 persistent heavy drinkers classified as having MetALD or ALD (n = 152) and abstinent patients with a history of excessive drinking (n = 151). Plasma CTSD levels of patients with MetALD/ALD without decompensation were compared with 40 healthy controls. Subsequently, the relationship between plasma CTSD levels and hepatic histological scores was established. Receiver-operating characteristic curves were generated to assess the precision of plasma CTSD levels in detecting MetALD/ALD. Lastly, plasma CTSD levels were compared between abstainers and drinkers.

Results: Plasma CTSD levels were higher in patients with MetALD/ALD compared to healthy controls. While hepatic disease parameters (AST/ALT ratio, liver stiffness measurement) were higher at advanced histopathological stages (assessed by liver biopsy), plasma CTSD levels were already elevated at early histopathological stages. Furthermore, combining plasma CTSD levels with liver stiffness measurement and AST/ALT ratio yielded enhanced diagnostic precision (AUC 0.872) in detecting MetALD/ALD in contrast to the utilization of CTSD alone (AUC 0.804). Plasma CTSD levels remained elevated in abstainers.

Conclusion: Elevated levels of CTSD in the circulation can serve as an early indicator of MetALD/ALD.

© 2024 The Author(s). Published by Elsevier B.V. on behalf of European Association for the Study of the Liver (EASL). This is an open access article under the CC BY license (http://creativecommons.org/licenses/by/4.0/).

Introduction

Alcohol-related liver disease (ALD) is the leading cause of liver disease-related morbidity and mortality worldwide. In 2017, there were an estimated 23.6 million cases of compensated cirrhosis and 2.5 million cases of decompensated cirrhosis attributable to alcohol use worldwide.[1] Moreover, according to a 2014 global report on non-communicable diseases by the World Health Organization, approximately 3.3 million deaths annually, representing 5.9% of global deaths, were attributed to excessive alcohol consumption. Among these deaths, over half were associated with non-communicable diseases, with 16% specifically linked to end-stage liver disease.[2,3] Relevantly, there has been a notable update in the terminology used to describe liver diseases caused by alcohol consumption. This update has led to the differentiation between alcohol-related liver disease (ALD) and metabolic dysfunction/alcohol-related liver disease (MetALD), with the primary distinction being based on the current level of alcohol consumption.[4]

From a pathological point of view, ongoing excessive drinking increases the likelihood of developing hepatic pathological events ranging from hepatic steatosis, hepatic inflammation and ballooning, fibrosis and cirrhosis, which can eventually lead to hepatocellular carcinoma (HCC) and ultimately liver failure.[5] Consistent with global alcohol consumption data, the prevalence of MetALD/ALD has remained stable over the past two decades, while the incidence of advanced stages of MetALD/ALD has shown a consistent increase.[6] Considering these findings, early detection of MetALD/ALD has become an increasingly urgent priority to prevent its progression to advanced stages.

General practitioners (GPs) play a crucial role in the identification and initiation of interventions for patients with MetALD/ALD. Several questionnaire-based studies have assessed GPs' recognition, attitudes, and interventions concerning excessive alcohol consumption and alcohol-related problems. The findings consistently indicate low levels of recognition and intervention by GPs regarding excessive alcohol consumption.[7,8] Fear of damaging the doctor-patient relationship, concerns about violating patient integrity, and reluctance to condemn excessive drinkers are some of the reasons why GPs may not screen for

* Corresponding author. Address: Department of Genetics and Cell Biology, Institute of Nutrition and Translational Research in Metabolism, Maastricht University, The Netherlands.
E-mail address: r.sverdlov@maastrichtuniversity.nl (R. Shiri-Sverdlov).
https://doi.org/10.1016/j.jhepr.2024.101117

alcohol use.[9] Furthermore, patients often conceal their drinking habits.[5,8] Consequently, a subset of patients with liver disease may go unnoticed, as they are not motivated to abstain from alcohol or seek treatment, allowing the disease to progress.[10] Although advanced hepatic pathological stages of ALD (fibrosis, cirrhosis) can be detected through non-invasive imaging techniques (Fibroscan) or liver biopsy, there are currently no accurate methods to diagnose early MetALD/ALD, posing challenges for timely detection.[11] Thus, there is an urgent need for new approaches to enable early diagnosis of MetALD/ALD in patients with chronic heavy alcohol consumption.

Accumulating evidence established the central role of lysosomal cathepsins in the development of low-grade chronic inflammation and related metabolic disturbances within the context of obesity-associated diseases.[12] Amongst cathepsins, cathepsin D (CTSD) has attracted increased attention in recent years due to its role in lysosomal cell death and the fact that it is one of the few cathepsins that shows some activity at neutral pH. Specifically, we showed that plasma CTSD levels correlated with different stages of diet-induced steatotic liver disease in adults[13] and even demonstrated that plasma CTSD levels were an early marker of diet-induced steatotic liver disease in children.[12] We subsequently demonstrated increased plasma levels and activity of CTSD in patients with metabolic dysfunction-associated steatohepatitis (MASH) and type 2 diabetes.[14] However, while *in vivo* and *in vitro* studies have demonstrated an association between hepatic changes in CTSD levels and alcohol intake,[15,16] to our knowledge, no study has ever investigated plasma CTSD levels in the context of MetALD/ALD.

For this purpose, we hypothesized that plasma CTSD levels can discriminate between healthy controls and patients with MetALD/ALD and, moreover, associate with early histopathological stages of MetALD/ALD. Therefore, plasma CTSD levels were quantified in both healthy controls and patients with MetALD/ALD and subsequently correlated with various hepatic histopathological indicators that were scored according to standardized criteria. Subsequently, the accuracy of plasma CTSD levels alone or in combination with known non-invasive markers of hepatic disease was investigated in patients with MetALD/ALD.

Materials and methods

Participant characteristics

We conducted a cross-sectional study of 303 individuals at risk of MetALD/ALD from the Region of Southern Denmark, classified as persistent heavy drinkers (n = 152) and abstinent patients with a history of excessive drinking (n = 151). The protocol was approved by the ethics committee of the Danish Data Protection Agency and the Region of Southern Denmark (S-20120071, S-20160021 and S-20160006G) and adheres to the 2013 Declaration of Helsinki. Results from the cohort have previously been published.[17–19] In short, patients were prospectively recruited between April 18, 2013 and September 17, 2018 from referrals to outpatient liver clinics or primary care. 27% of patients in this cohort had advanced fibrosis. Initially, persistent heavy drinking was defined as at least 1 year of drinking above the limits for harmful drinking as defined by the Danish health authorities (≥14 units/week for females and ≥21 units per week for males).[20] In 2016, this protocol was revised to require at least 5 years of heavy drinking based on the previously indicated criteria. Inclusion criteria, in addition to alcohol history, were age 18-75 years, and informed consent for a liver biopsy.[20] We excluded patients with decompensation at baseline.

For comparison, we included 40 healthy controls matched for age and gender with patients at risk of MetALD/ALD. Recruitment and inclusion of healthy controls are described elsewhere in detail.[21,22] The inclusion criterion for healthy controls was age 40-75 years. Exclusion criteria included: 1) medication use, prescription or otherwise at the time of inclusion; 2) suffering from a chronic disease; 3) antibiotic use 6 months before the start of inclusion; 4) reported alcohol intake above the low-risk limit of 7 units of alcohol per week for females and 14 units per week for males, or binge drinking ≥5 units at one event.

Histology

An experienced pathologist (blinded to clinical data) scored all liver biopsies according to NAFLD activity score (NAS) of the Clinical Research Network for steatosis (0-3), ballooning (0-2), lobular inflammation (0-3) and fibrosis (0-4).[17,20] The sum of ballooning and lobular inflammation constitutes the composite score for inflammatory activity (0-5). A composite NAS score consists of the sum of steatosis, ballooning, and lobular inflammation, the histological features of active injury that are potentially reversible in the short term (0-8).

History of alcohol intake

Patients reported their alcohol use history on a standard questionnaire, detailing duration and degree of overuse, peak drinking, current alcohol intake, and, if abstaining from alcohol at the time of inclusion, the duration of abstinence.[17] Abstinence was defined as not drinking any alcoholic beverages for at least 1 week.[20]

Biochemical analyses, liver fibrosis and liver biopsy

We performed all investigations and blood sampling for biobanking within 1 week of inclusion. After overnight fasting, we collected venous blood. Fasting blood glucose, insulin, homeostasis model assessment of insulin resistance, glycosylated hemoglobin, plasma triglycerides, HDL and LDL, alanine aminotransferase (ALT), aspartate aminotransferase (AST), model for end-stage liver disease, and C-reactive protein were determined using routine analyses.[20]

The enhanced liver fibrosis test (Siemens Healthcare Diagnostics Inc., Tarrytown, NY) was measured on an Advia Centaur XP (Siemens Healthcare Diagnostics Inc.) according to the manufacturer's instructions. The coefficient of variation estimated from the assay controls with three different concentrations ranged from 4.1% to 6.1% for hyaluronic acid, from 4.0% to 5.4% for the N-terminal pro-peptide of collagen type III, and from 2.5% to 2.9% for tissue inhibitor of metalloproteinase-1. A cut-off of 10.5 was used, convenience adjusted from 10.51 as recommended by the National Institute for Health and Care Excellence for advanced fibrosis in NAFLD.[23,24] The manufacturer-recommended 9.8 and 7.7 cut-offs were considered; the first to diagnose advanced fibrosis (≥F3), the latter to exclude significant fibrosis (≥F2).[25,26] One

experienced nurse operator (>500 scans) determined liver stiffness measurement (LSM) by transient elastography (TE) using a FibroScan 502 Touch (Echosens, Paris, France) according to standard.[27,28] An ultrasound-trained investigator (>300 scans per year) performed abdominal ultrasonography and 2-dimensional shear-wave elastography with the Aixplorer system (Supersonic Imagine, Aix-en-Provence, France) according to published standards.[27,29] As recommended by the 2015 Baveno VI consensus paper, we considered LSM ≥15.0 kPa as optimal for diagnosing advanced fibrosis.[30] In *post hoc* analyses, we checked whether the 15.0 kPa cut-off was appropriate for our cohort, given that ALD may yield higher liver stiffness than other etiologies.[31] The optimal cut-off for ≥F3 for LSM was 15.5. We also evaluated our previously published optimal cut-offs for cirrhosis; 19.7 kPa for LSM.

Finally, a percutaneous suction needle liver biopsy was performed in the same intercostal space as the elastography (17 G Menghini needle; Hepafix, Braun, Germany). An experienced liver pathologist assigned Kleiner fibrosis stage while blinded to all other study data (previously reported, Kappa 0.71 for intra-observer variance).[32] We considered a biopsy to be of adequate quality if it had a length of at least 10 mm and at least six portal tracts, or presence of regenerative nodules. In 2016, we changed the study protocol, since no patient with LSM <6.0 kPa had advanced fibrosis. We therefore refrained from a liver biopsy in 96 patients with LSM below 6.0 kPa. Subclassifications according to the new nomenclature were made as described before.[33]

Human cathepsin D ELISA[12]

Human plasma was diluted 200x based on a pool test and CTSD levels were determined by means of the CTSD ELISA according to the manufacturer's protocol (USCN Life Science Inc., Wuhan, China). The absorbance was measured on a Benchmark 550 microplate reader (Bio-Rad, Hercules, CA, USA); the detection limit ranged from approximately 46.88 to 3,000 pg/ml. CTSD measurements were performed blinded to the histological findings of the study participants.

Statistical analyses

Statistical analyses were performed using SPSS 27.0 Statistics for Windows (IBM, Armonk, N.Y., USA), MedCalc (Version 20.106) and GraphPad Prism 9.0 for Microsoft Windows. The differences in participants' characteristics were tested using an independent sample t-test. Further, all analyses were conducted using non-parametric tests as the data were not normally distributed. The data were expressed as median ± interquartile range and considered significant at p <0.05. Correlation coefficients were calculated to determine simple associations between plasma CTSD levels and hepatic disease-related features, as well as plasma LDL cholesterol and plasma triglycerides. Subsequently, multiple linear regression analyses were performed, in which plasma CTSD levels served as a dependent variable and other parameters as independent variables, resulting in model 1 (simple regression), model 2 (model 1 + adjustment for age), model 3 (model 2 + adjustment for BMI) and model 4 (model 3 + adjustment for sex). p values <0.05 were considered statistically significant. Areas under the receiver-operating characteristic curve (AUCs) were generated in SPSS to investigate the diagnostic accuracy of plasma CTSD levels alone or in combination with current diagnostic markers for MetALD/ALD. The cut-off values were derived from the AUC and calculated using Youden's index formula. The comparison between two AUCs was determined as described by DeLong *et al.*[34] using MedCalc. A significance level of p <0.05 was deemed significant.

Results

Clinical characteristics of individuals

A total of 345 individuals, including 152 patients with MetALD/ALD, 151 abstainers and 40 healthy controls were enrolled in the study. The clinical characteristics of these individuals are presented in Table 1. Further, AST/ALT ratio, LSM and plasma CTSD levels were increased in patients with MetALD/ALD compared to healthy controls (Fig. 1).

Table 1. Baseline clinical characteristics.

Parameters	Healthy controls (n = 40)	MetALD/ALD (n = 152)	Abstainer (n = 151)	Total (n = 345)	p value (MetALD/ALD *vs.* healthy controls)
Gender (F/M)	9/31	31/121	51/140	82/263	0.772
Age (yr)	53±1.6	57±0.9	54±0.9	55.04±0.59	0.070
BMI (kg/m^2)	26±0.5	28±0.5	27±0.4	27.18±0.29	0.031*
HOMA-IR	2.8±0.4	6.4±0.9	4.5±0.4	5.18±0.44	0.000*
Insulin (pmol/L)	69.4±8.0	127.7±13.4	101.4±8.7	109.90±7.15	0.000*
Fasting glucose (mmol/L)	5.9±0.2	6.7±0.2	6.9±0.2	6.82±0.12	0.000*
Hemoglobin A1c (mmol/mol)	37±1.0	37±0.9	39±0.9	37.96±0.56	0.927
LDL cholesterol (mmol/L)	3.3±0.1	2.8±0.1	3.2±0.1	3.03±0.06	0.003*
HDL cholesterol (mmol/L)	1.3±0.08	1.5±0.05	1.3±0.03	1.37±0.03	0.119
Triglyceride (mmol/L)	1.3±0.1	1.9±0.2	1.4±0.1	1.60±0.07	0.001*
CAP (dB/m)	270±9	307±7	257±6	281.58±4.38	0.002*
ALT (U/L)	31±3	48±3	33±2	39.62±1.85	0.000*
AST (U/L)	28±2	59±4	38±3	46.33±2.15	0.000*
AST/ALT ratio	1.01±0.06	1.46±0.07	1.28±0.05	15.18±0.97	0.000*
LSM (kPa)	7.0±1.5	16.7±1.5	15.9±1.5	7.18±0.10	0.000*
MELD	6.7±0.1	7.4±0.2	7.1±0.1	1.34±0.04	0.005*
CRP (mg/L)	3.2±0.5	8.1±1.7	6.5±1.1	6.89±0.93	0.007*
Plasma CTSD levels (pg/ml)	22,858.54±1,059.15	33,766.34±980.38	41,501.97±1,218.73	35,939.20±766.94	0.000*

Data are represented as mean±SEM. Data are statistically analyzed by performing an independent sample t-test using SPSS software, version 27.0 (SPSS, Chicago, IL, USA).
* p <0.05 is statistically significant.
ALD, alcohol-related liver disease; ALT, alanine aminotransferase; AST, aspartate aminotransferase; CAP, controlled attenuation parameter; CRP, C-reactive protein; CTSD, cathepsin D; HOMA-IR, homeostasis model assessment of insulin resistance; LSM, liver stiffness measurement; MELD, model for end-stage liver disease; MetALD, metabolic dysfunction and alcohol-related liver disease.

The elevation of plasma CTSD levels at early histopathological stages of MetALD/ALD relates to changes in lipid metabolism

Next, we investigated the relationship of hepatic histopathological events, assessed according to the NAS system,[17] with AST/ALT ratio, LSM and plasma CTSD levels. Although the NAS steatosis score exhibited no variations in AST/ALT ratio (Fig. 2A), LSM showed a gradual increase with higher NAS steatosis scores (Fig. 2B). Conversely, the mean absolute levels of plasma CTSD were highest at the lowest NAS steatosis score and remained rather stable when NAS steatosis scores increased (Fig. 2C). Furthermore, the NAS inflammation score confirmed these findings: while the existing hepatic disease markers AST/ALT ratio and LSM significantly increased at advanced NAS inflammation scores (Fig. 2D,E), plasma CTSD levels were highest at the lowest NAS inflammation scores (Fig. 2F). Similar observations were also made in patients with MetALD/ALD categorized according to the total NAS score, showing increased AST/ALT ratio and LSM only at advanced stages compared to healthy controls (Fig. S1A,B). In contrast, plasma CTSD levels increased in patients with MetALD/ALD with the lowest NAS scores and remained stable upon increasing NAS scores (Fig. S1C).

In line with these findings, AST/ALT ratio and LSM were only increased upon advanced hepatic fibrosis stages (Fig. 2G,H), while a significant elevation of plasma CTSD levels was already apparent in the early stages of hepatic fibrosis (Fig. 2I). Moreover, plasma CTSD levels increased among patients with MetALD/ALD exhibiting an AST/ALT ratio <2 compared to healthy controls (Fig. 3). This elevation decreased in comparison to patients with AST/ALT levels >2 (Fig. 3).

To explore the underlying mechanisms that explain the early increase of plasma CTSD levels in these patients with MetALD/ALD, we described the baseline characteristics of the healthy controls and score 0 MetALD/ALD patients according to the NAS classification (Table S1). This classification unveiled that, next to plasma CTSD levels, plasma triglycerides and plasma LDL cholesterol (the latter only a trend) are also different between healthy controls and score 0 MetALD/ALD patients. To subsequently test whether the increase in plasma CTSD levels in the score 0 MetALD/ALD patients (compared to healthy controls) associates with the observed changes in plasma triglycerides and/or LDL cholesterol levels, we performed a multiple linear regression analysis combining the healthy controls and score 0 MetALD/ALD patients. While plasma CTSD levels did not associate with plasma LDL cholesterol levels, a positive independent association with plasma triglycerides was found (Model 1: standardized β, 0.286; p = 0.030), also after adjustment for age (Model 2: standardized β, 0.270; p = 0.034), BMI (Model 3: standardized β, 0.293; p = 0.026) and sex (Model 4: standardized β, 0.285; p = 0.034) (Table S2). Together, these findings indicate that plasma CTSD levels associate with early stages of MetALD/ALD and suggest that the observed increase in plasma CTSD levels is related to changes in lipid metabolism.

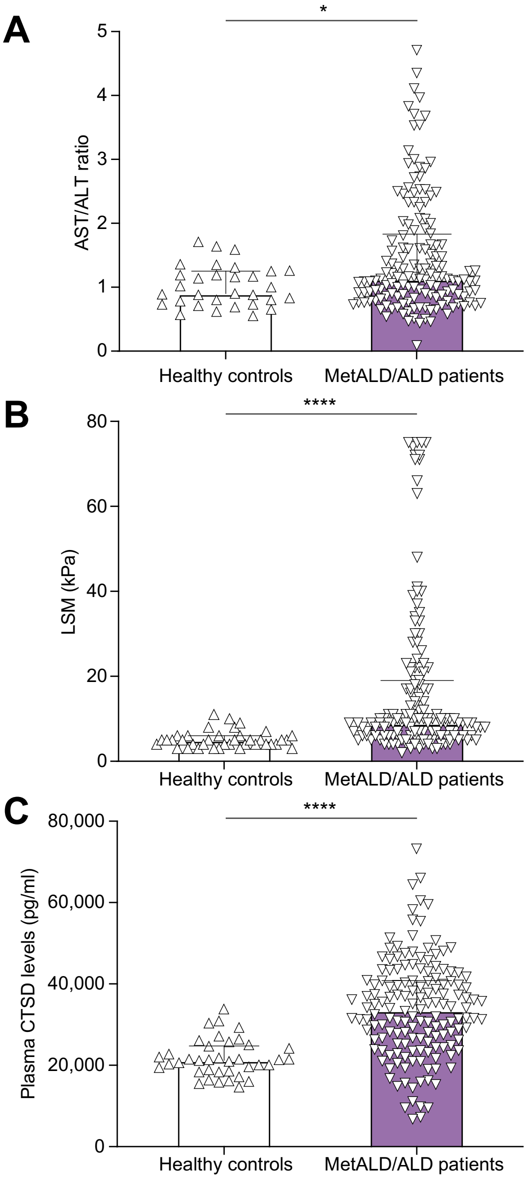

Fig. 1. Plasma CTSD levels and hepatic disease markers in MetALD/ALD patients and healthy controls. (A) AST/ALT ratio of healthy controls *vs*. patients with MetALD/ALD (healthy control, n = 31; MetALD/ALD, n = 148). (B) LSM of healthy controls *vs.* patients with MetALD/ALD (healthy control, n = 38; MetALD/ALD, n = 145). (C) CTSD in the plasma of healthy controls *vs.* patients with MetALD/ALD (healthy control, n = 38; MetALD/ALD, n = 152). *p <0.05, ****p <0.0001 (Mann-Whitney test). All error bars are median with interquartile range. ALD, alcohol-related liver disease; ALT, alanine aminotransferase; AST, aspartate aminotransferase; CTSD, cathepsin D; LSM, liver stiffness measurement; MetALD, metabolic dysfunction and alcohol-related liver disease.

Plasma CTSD levels correlate with hepatic disease determinants in patients with MetALD/ALD

Considering its association with MetALD/ALD, we next examined the liver specificity of plasma CTSD levels by correlating their values with hepatic disease markers. In accordance with the absence of alterations in fibrosis staging, plasma CTSD levels correlated in a weak, inverse manner with hepatic fibrosis indicators including ELF and LSM (Fig. 4A; r = -0.1493, p = 0.07, Fig. 4B; r = -0.2954, p <0.001). Additionally, in patients with MetALD/ALD, plasma CTSD levels were inversely associated with AST, AST/ALT ratio and controlled attenuation parameter (Fig. S2A; r = -0.2592, p = 0.001, Fig. S2B; r = -0.2895, p <0.001, Fig. S2C; r = -0.1903, p = 0.07, respectively).

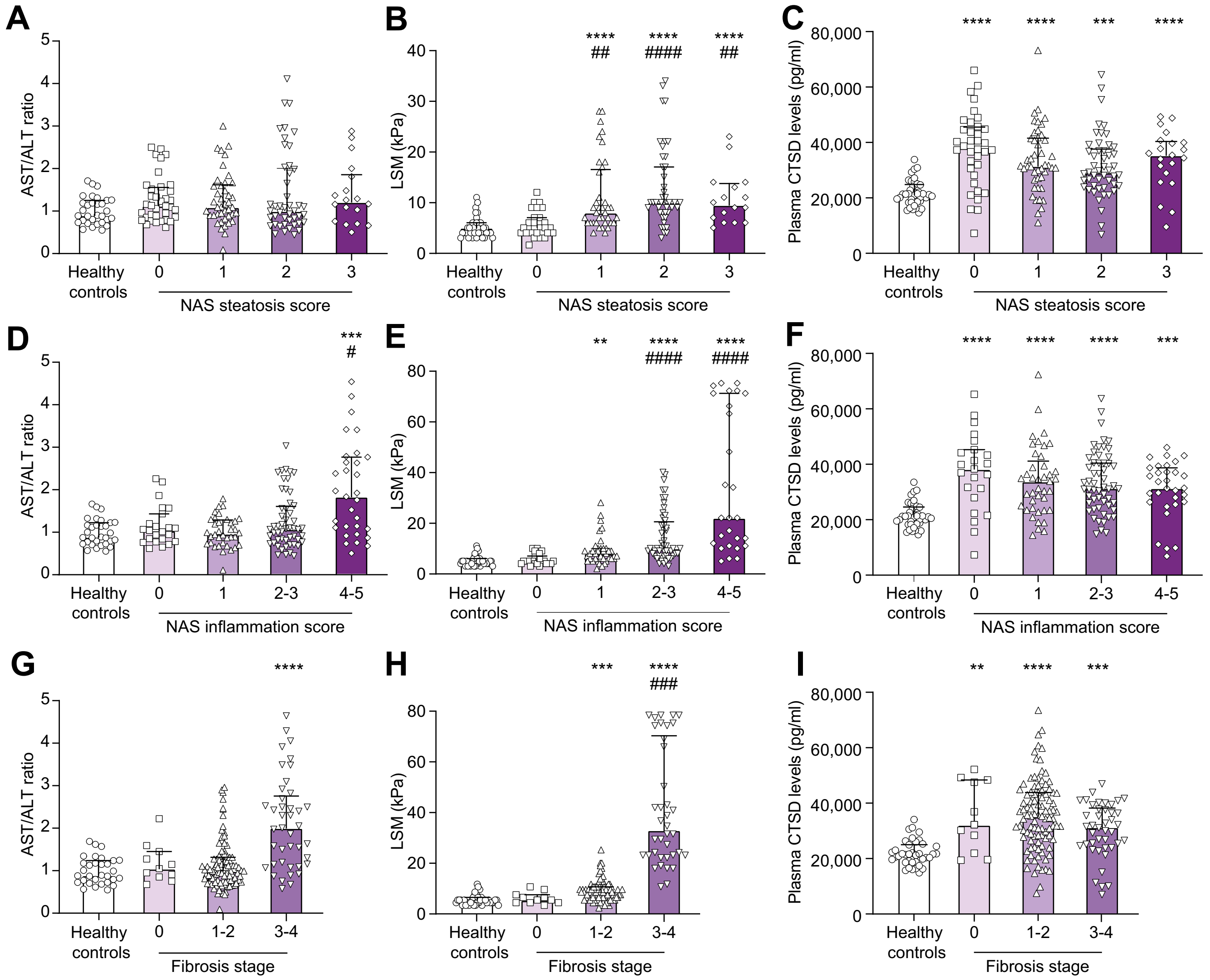

Fig. 2. Plasma CTSD levels and hepatic disease markers increased in patients with MetALD/ALD classified according to hepatic histological criteria. AST/ALT ratio, LSM and CTSD levels were analyzed in healthy controls (n = 40) and patients with MetALD/ALD (n = 152) and classified by liver biopsy-proven NAS steatosis score (0-3) (A-C), NAS inflamamtion score (including ballooning (0-2) and lobular inflammation (0-3)) (D-E), and fibrosis stage (0-4) (G-I) according to NAS. *p <0.05, **p <0.01, ***p <0.001, ****p <0.0001 compared to healthy controls and $^{\#}p$ <0.05, $^{\#\#}p$ <0.01, $^{\#\#\#}p$ <0.001, $^{\#\#\#\#}p$ <0.0001 compared to score 0 MetALD/ALD patients (Kruskal-Wallis test). All error bars are median with interquartile range. ALD, alcohol-related liver disease; ALT, alanine aminotransferase; AST, aspartate aminotransferase; CTSD, cathepsin D; LSM, liver stiffness measurement; MetALD, metabolic dysfunction and alcohol-related liver disease; NAS, NAFLD activity score.

Moreover, multiple linear regression analyses were performed to further evaluate whether plasma CTSD levels were independently associated with these hepatic disease parameters. As represented in Table S3, we found that plasma CTSD levels were independently associated with LSM, AST/ALT ratio, and AST in patients with MetALD/ALD, whereas the correlation between CTSD and ELF was dependent on age.

Plasma CTSD levels hold a better predictive value than AST/ALT ratio and LSM for detecting MetALD/ALD

We next compared the clinical potential of plasma CTSD levels, AST/ALT ratio and LSM for diagnosing MetALD/ALD by generating receiver-operating characteristic curves. When comparing patients with MetALD/ALD to healthy controls, plasma CTSD levels demonstrated the highest AUC (=0.804), followed by LSM (AUC = 0.764) and AST/ALT ratio (AUC = 0.615) (Fig. 5). Additional statistical analysis revealed that plasma CTSD levels significantly outperformed AST/ALT ratio (p = 0.0072), while no difference towards outperformance was observed compared to LSM (p = 0.5070) (Table S4). Also, a significant difference was observed between the AUCs of AST/ALT ratio and LSM (p = 0.0416) (Table S4). The diagnostic performances of CTSD levels, AST/ALT ratio, and LSM in diagnosing MetALD/ALD revealed optimal cut-off values of ≥21,933.10 pg/ml for CTSD levels, ≥1.37 for AST/ALT ratio, and ≥6.5 kPa for LSM (Table S5). Plasma CTSD levels demonstrated the highest sensitivity (87%) compared to AST/ALT ratio and LSM, whereas the specificity (68%) was lower than LSM (74%) and AST/ALT ratio (90%). Positive and negative predictive values were 92% and 53%,

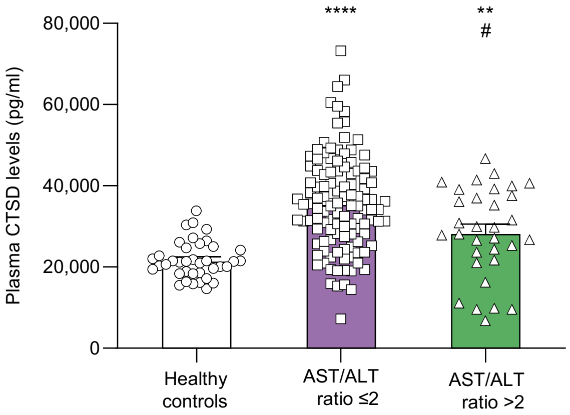

Fig. 3. Plasma CTSD levels in patients with MetALD/ALD stratified by AST/ALT ratio at cut-off level 2. $^{**}p$ <0.01, $^{****}p$ <0.0001 compared to healthy controls and $^{\#}p$ <0.05 compared to MetALD/ALD patients with AST/ALT ratio ≤2 (indicated in graph as 'AST/ALT ratio ≤2') (Kruskal-Wallis test). All error bars are median with interquartile range. (healthy controls, n = 38; AST/ALT ratio ≤2, n = 116; AST/ALT ratio >2, n = 32). ALD, alcohol-related liver disease; ALT, alanine aminotransferase; AST, aspartate aminotransferase; CTSD, cathepsin D; MetALD, metabolic dysfunction and alcohol-related liver disease.

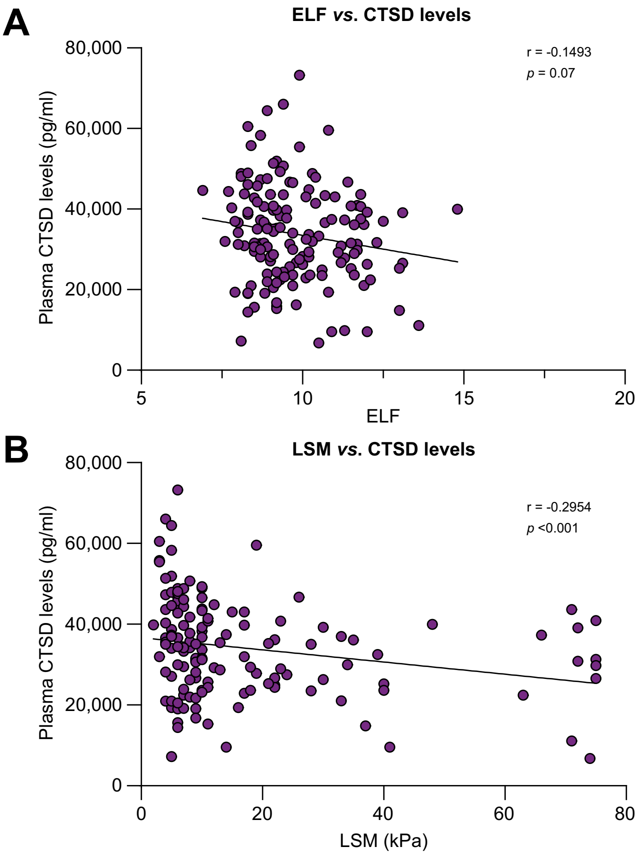

Fig. 4. Plasma CTSD levels correlate with hepatic fibrosis determinants in patients with MetALD/ALD. (A) Plasma CTSD levels *vs.* ELF (n = 152) and (B) plasma CTSD levels *vs.* LSM (n = 145). Spearman's correlation was performed. p <0.05 was considered statistically significant. ALD, alcohol-related liver disease; CTSD, cathepsin D; ELF, enhance liver fibrosis; LSM, liver stiffness measurement; MetALD, metabolic dysfunction and alcohol-related liver disease.

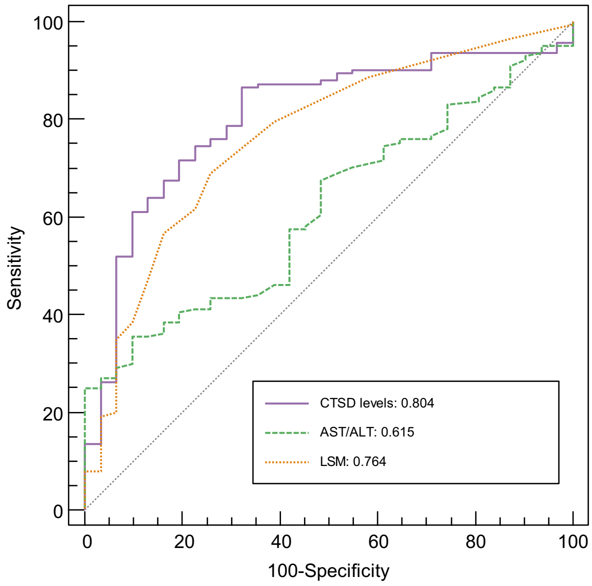

Fig. 5. The diagnostic value of plasma CTSD, AST/ALT ratio and LSM. Receiver-operating characteristic curve analysis was performed to evaluate the AUC of plasma CTSD, AST/ALT ratio or LSM to predict MetALD/ALD. ALD, alcohol-related liver disease; ALT, alanine aminotransferase; AST, aspartate aminotransferase; CTSD, cathepsin D; LSM, liver stiffness measurement; MetALD, metabolic dysfunction and alcohol-related liver disease.

while positive likelihood ratio (LR+) and negative likelihood ratio (LR-) were 2.7 and 0.2 for CTSD levels (Table S5). The diagnostic accuracy of CTSD levels was 83%, while LSM and AST/ALT ratio only reached diagnostic accuracies of 70% and 45%, respectively (Table S5).

Adding plasma CTSD levels to AST/ALT ratio and LSM improves diagnostic accuracy for MetALD/ALD prediction

To explore whether the addition of plasma CTSD levels to the existing hepatic disease parameters would benefit MetALD/ALD prediction, receiver-operating characteristic curves of AST/ALT ratio and LSM with or without plasma CTSD levels were plotted. The addition of plasma CTSD to AST/ALT ratio (AUC = 0.858; p <0.0001) and LSM (AUC = 0.857; p = 0.0258) significantly increased the diagnostic accuracy of both hepatic disease parameters (Fig. 6 and Table S6). Moreover, combining all three parameters (CTSD, ALT/AST and LSM) resulted in the highest AUC (=0.872) and confirmed that the addition of plasma CTSD levels improved the AUC compared to the combination of AST/ALT ratio and LSM (p = 0.0002) (Fig. 6 and Table S6).

Similar to the description of the single parameters, the diagnostic performances of the combination of CTSD, AST/ALT ratio and LSM in the prediction of MetALD/ALD are depicted in Table S7. Combining CTSD, AST/ALT ratio and LSM to predict MetALD/ALD resulted in a sensitivity of 85% and a specificity of 84%. Positive predictive value and negative predictive value were 96% and 55%, while LR+ and LR-were 5.3 and 0.2, respectively (Table S7). Diagnostic accuracy was 85%, which was the highest amongst all combinations (Table S7). Altogether, these results illustrate the value of plasma CTSD levels for MetALD/ALD prediction when combined with two other hepatic disease parameters.

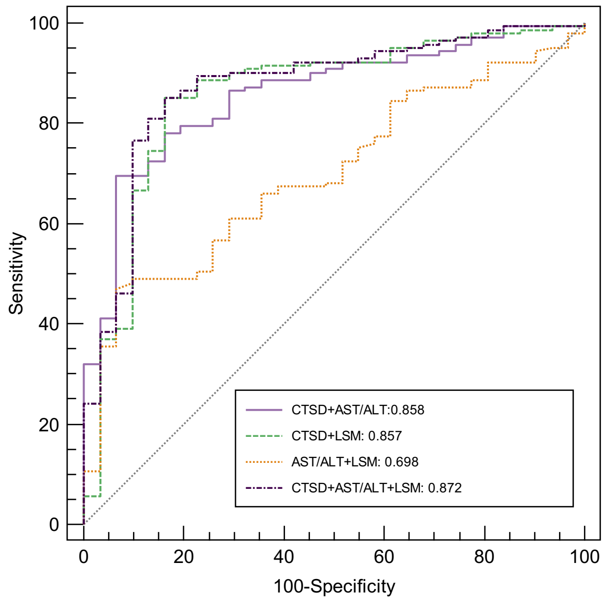

Fig. 6. The diagnostic value of adding CTSD to AST/ALT ratio and LSM. Receiver-operating characteristic curve analysis was performed to evaluate the AUC of using the combination of CTSD levels with AST/ALT ratio and LSM to predict MetALD/ALD. ALD, alcohol-related liver disease; ALT, alanine aminotransferase; AST, aspartate aminotransferase; CTSD, cathepsin D; LSM, liver stiffness measurement; MetALD, metabolic dysfunction and alcohol-related liver disease.

Plasma CTSD levels remain elevated after self-reported abstinence of alcohol consumption

In order to explore any potential decrease in plasma CTSD levels following cessation of alcohol consumption, we categorized individuals into three groups: healthy controls, abstinent individuals (with durations of abstinence ranging from less than 1 year to over 30 years) (Table S8), and current drinkers. Measurement of plasma CTSD levels showed a significant increase in drinking individuals when compared to healthy controls, which remained elevated even after abstinence (Fig. 7).

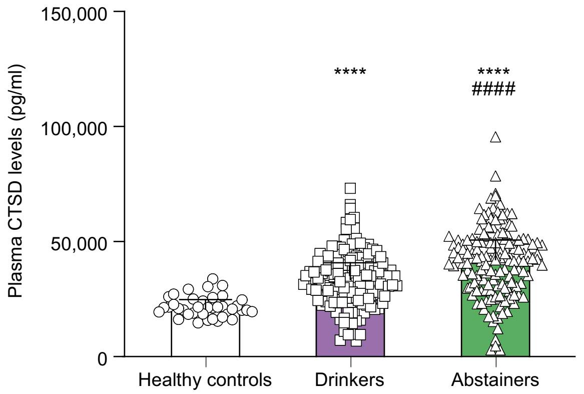

Fig. 7. Plasma CTSD levels in drinkers and abstinent individuals. $^{****}p$ <0.0001 compared to healthy controls, $^{\#\#\#\#}p$ <0.0001 compared to drinkers (Kruskal-Wallis test). All error bars are median with interquartile range. (healthy controls, n = 38; drinking individuals, n = 152; abstinent individuals, n = 151).

Moreover, abstainers who had self-reportedly refrained from alcohol consumption for less than 1 year exhibited notably higher CTSD levels compared to individuals currently engaged in drinking (Fig. S3).

Discussion

Individuals who consistently consume large quantities of alcoholic beverages are at a high risk of developing MetALD/ALD, which often goes undetected until advanced stages. Hence, novel, early biomarkers are required to identify MetALD/ALD before it advances to later stages. In this study, we propose plasma CTSD levels as an early indicator of MetALD/ALD.

In our previous work, we established a connection between CTSD and hepatic inflammation in adult patients with MASH.[13] In the present study, we identified an association between plasma CTSD levels and MetALD/ALD. Moreover, in alignment with our prior observations showing elevated CTSD levels during the early histopathological stages in patients with MASH, we identified a parallel trend in the MetALD/ALD patient population. These observations suggest that CTSD may serve as an early biomarker for hepatic disease but is likely not exclusive to MetALD/ALD.

Lysosomes and lysosomal enzymes are considered to play important roles in alcohol-induced disorders, such as ALD, HCC, and alcohol-induced pancreatitis.[35] In our study, we demonstrated that patients with MetALD/ALD exhibit consistently high levels of plasma CTSD, particularly notable during the early histopathological stages of disease. Alcohol has been shown to damage lysosomal function and induce the release of exosomes from liver cells.[36] Likewise, Babuta *et al.* have shown, in both mouse models of alcohol-related liver disease and alcohol-related hepatitis, as well as in human ALD livers, that the rise in exosome production triggered by alcohol is associated with the impairment of autophagy and lysosomal function.[37] This evidence, in conjunction with the finding that CTSD has been detected in hepatocyte-derived exosomes,[38] suggests that elevated plasma CTSD levels in patients with MetALD/ALD may serve as a signal for alcohol-induced hepatic injury and/or potentially intercellular communication in response to alcohol exposure. Given the central role of the liver in lipid metabolism (*de novo* lipogenesis and catabolism), the hepatocyte-derived signal hypothesis of CTSD is further corroborated by our finding that plasma CTSD levels independently associate with plasma triglyceride levels when combining the healthy controls and score 0 MetALD/ALD patients. This finding is also in line with previous observations of our group showing that plasma triglycerides as well as CTSD levels were elevated in patients with MASH, though no independent association was explored in the latter publication.[13] Regardless of lipid metabolism, in the current study, we also demonstrate an independent association between plasma CTSD levels and hepatic disease markers (LSM, AST/ALT ratio and AST). All these observations therefore add substantial fuel to the hypothesis that the observed increased plasma levels of CTSD are derived from the liver and are due to a direct effect of alcohol on hepatic cellular physiology and metabolism.

Yet, it is imperative to acknowledge that extrahepatic sources potentially influencing alcohol-induced elevations of plasma CTSD levels cannot be disregarded and are even probable due to the systemic effect of alcohol intake.[39] For instance, macrophages (among other immune cells) carry

a large amount of proteolytic enzymes that are released upon a plethora of stimuli, including alcohol.[40,41] Furthermore, although alcohol has been linked to neuro-inflammation[42] and toxicity,[43] CTSD has been implicated in neurodegenerative processes observed in conditions such as Alzheimer's and Parkinson's disease.[44,45] This suggests that CTSD levels may also be influenced by alcohol-induced neurotoxicity.

While the liver biopsy is still considered the gold standard to detect ALD,[46] its diagnosis typically relies on a comprehensive evaluation encompassing patient history, clinical examination and non-invasive methods that include laboratory tests (*e.g.* AST/ALT ratio, ELF) and imaging techniques (*e.g*. LSM with FibroScan). However, this diagnostic process is challenged by the possibility of patients providing inaccurate information about their alcohol consumption. Furthermore, while the current laboratory and imaging markers have good predictive power for advanced stages of ALD,[46] they are generally not considered as appropriate markers for initial stages of MetALD/ALD. These concepts are confirmed by our data showing that AST/ALT ratio and LSM were only increased at advanced histopathological stages of MetALD/ALD. In contrast, plasma CTSD levels increased at early histopathological stages and moreover, outperformed AST/ALT ratio (AUC: 0.615; accuracy: 45%) and LSM (AUC: 0.764; accuracy: 70%) for the diagnosis of MetALD/ALD. Moreover, addition of CTSD to any of the existing markers also improved its diagnostic accuracy, underscoring the diagnostic potential of CTSD for MetALD/ALD. However, despite CTSD achieving the highest LR+ and lowest LR-values, none of these values met the conventional thresholds of LR+ ≥10 and LR- ≤0.1, which are typically indicative of evidence supporting either the inclusion or exclusion of a diagnosis.[47] Therefore, while our data demonstrate that plasma CTSD levels outperform AST/ALT ratio and LSM to predict MetALD/ALD, it is still important to exert caution before asserting CTSD as the definitive biomarker for diagnosing MetALD/ALD.

In contrast to our expectations, plasma CTSD levels persisted at elevated levels following abstinence among previously persistent heavy drinkers. This finding contradicts the observations from a previous study performed by our group that showed a reduction of plasma CTSD upon regression of MASH due to surgical intervention.[13] A potential explanation for this apparent difference is that, unlike diet-induced liver injury, alcohol-induced injury exerts a more profound cellular impact on the liver and may not be completely reversible even with abstinence from alcohol. However, it is worth noting that within this cohort, there are no markers available for monitoring alcohol consumption. Consequently, there is a possibility that patients inaccurately reported the duration of their abstinence,[48] influencing the interpretation of these findings. As a result, multiple uncertain factors must be considered when analyzing the sustained elevation of plasma CTSD levels after abstinence. Therefore, further investigation in an appropriate setting appears necessary to explore the time-dependent influence of alcohol abstinence on plasma CTSD levels.

One relevant limitation of this study is the inability to assess the actual impact of MetALD/ALD regression on CTSD levels due to a lack of plasma CTSD level measurements before and after individuals abstained from alcohol, as well as the absence of timely biochemical tests to evaluate their history of alcohol intake. Another limitation relates to the standard interval of our cohort of abstainers. Although we have different intervals (less than 1 year, 1-5 years, 6-10 years, etc.), the described periods are an approximation rather than exact duration of abstinence. The last limitation of this study, next to the small sample size of the healthy controls, is the mono-ethnic nature of this cohort. It is known that ethnicity is one of the major factors impacting the age of onset and severity of MetALD/ALD.[49] In addition, ethnicity affects the accuracy of biomarkers for determining MetALD/ALD; for instance, AST levels are 2-fold higher in African-American and Hispanic individuals compared with Caucasian, non-Hispanic Americans.[49] These findings indicate that ethnicity plays a role in clinical relevance, highlighting the need for future studies to validate CTSD's utility in MetALD/ALD across various ethnic populations.

In conclusion, in the current study, we demonstrated for the first time that elevated levels of plasma CTSD can serve as an early indicator of MetALD/ALD. Hence, our observations imply a diagnostic role for CTSD. Based on our past findings, therapies aimed at targeting CTSD should be further investigated in the context of ALD.

Affiliations

[1]*Department of Genetics and Cell Biology, Institute of Nutrition and Translational Research in Metabolism, Maastricht University, the Netherlands;* [2]*Center for Liver Research, Odense University Hospital and University of Southern Denmark, Kloevervaenget 10, entrance 112, DK-5000 Odense, Denmark;* [3]*Department of Internal Medicine Division of Gastroenterology and Hepatology, Maastricht University Medical Centre, Maastricht, The Netherlands;* [4]*Department of Laboratory Medicine, Medical University of Vienna, 1090 Vienna, Austria;* [5]*Laboratory of Hepatology, Department of Chronic Diseases and Metabolism, KU Leuven, Belgium; Department of Gastroenterology & Hepatology, University Hospitals Leuven, Leuven, Belgium*

Abbreviations

ALD, alcohol-related liver disease; ALT, alanine aminotransferase; AST, aspartate aminotransferase; CTSD, cathepsin D; ELF, enhanced liver fibrosis; LR, likelihood ratio; LSM, liver stiffness measurement; MASH, metabolic dysfunction-associated steatohepatitis; MetALD, metabolic dysfunction and alcohol-related liver disease; NAS, NAFLD activity score; TE, transient elastography.

Financial support

This study was funded by the European Union's Horizon 2020 research and innovation program (GALAXY, grant number 668031), and from the Novo Nordisk Foundation (MicrobLiver, grant number NNF15OC0016692; DECIDE, grant number NNF20OC0059393). RS was funded by TKI-LSH (grant no 40-41200-98-9306). ML was supported by the Chinese Scholarship Council with file number CSC202007550013.

Conflict of interest

AK has served as speaker for Novo Nordisk, Norgine, Siemens and Nordic Bioscience and participated in advisory boards for Siemens, Boehringer Ingelheim and Novo Nordisk, all outside the submitted work. Research support; Norgine, Siemens, Nordic Bioscience, Astra, Echosense. Board member and co-founder Evido. GK reports fee's from Gablon Pharma. MK reports a speaker's fee from Siemens Healthcare. MT reports speaker's fee from Echosens, Siemens Healthcare, Takeda, Norgine, Madrigal, Tillotts Pharma, and advisory fee from Boehringer Ingelheim, Astra Zeneca, Novo Nordisk and GSK. She is board member for Alcohol & Society (NGO) and co-founder and board member for Evido. Other authors have no relevant financial or non-financial interests to disclose.

Please refer to the accompanying ICMJE disclosure forms for further details.

Authors' contributions

RS-S, MT and TH were responsible for concept and design of study and final review of manuscript. ML, TH, AB, and DM were responsible for drafting of manuscript, data collection, statistical analysis, and critical review of manuscript. MEI, MK, GHK, TH, JV, and AAK were responsible for helping data collection and analysis. All authors contributed to the article and approved the submitted version.

Data availability statement

The datasets used and/or analyzed during the current study are available from the corresponding author on reasonable request.

Acknowledgements

We would like to acknowledge the support of the present and past members from Prof. Dr. Ronit Shiri-Sverdlov's team and Prof. dr. Maja Thiele for their help in data collection support and all subjects for their participation in this study.

Supplementary data

Supplementary data to this article can be found online at https://doi.org/10.1016/j.jhepr.2024.101117.

References

Author names in bold designate shared co-first authorship

[1] **Zhang X**, **Zhang X**, Liu M, et al. Global, regional, and national burden of cirrhosis and other chronic liver diseases due to alcohol use, 1990–2019: a systematic analysis for the Global Burden of Disease study 2019. BMC Gastroenterol 2022;22:484.
[2] Thursz M, Gual A, Lackner C, et al. Liver EaftSot EASL clinical practice guidelines: management of alcohol-related liver disease. J Hepatol 2018;69:154–181.
[3] World Health O. Global status report on noncommunicable diseases 2014. Geneva: World Health Organization; 2014.
[4] Rinella ME, Lazarus JV, Ratziu V, et al. A multisociety Delphi consensus statement on new fatty liver disease nomenclature. J Hepatol 2023;79:1542–1556.
[5] Torruellas C, French SW, Medici V. Diagnosis of alcoholic liver disease. World J Gastroenterol 2014;20:11684–11699.
[6] Wong T, Dang K, Ladhani S, et al. Prevalence of alcoholic fatty liver disease among adults in the United States, 2001-2016. JAMA 2019;321:1723–1725.
[7] Kaner EFS, Heather N, Mcavoy BR, et al. Intervention for excessive alcohol consumption in primary health care: attitudes and practices of English general practitioners. Alcohol Alcohol 1999;34:559–566.
[8] Coste S, Gimenez L, Comes A, et al. Discussing alcohol use with the GP: a qualitative study. BJGP Open 2020;4:bjgpopen20X101029.
[9] Arborelius E, Thakker KD. Why is it so difficult for general practitioners to discuss alcohol with patients? Fam Pract 1995;12:419–422.
[10] Mellinger JL, Scott Winder G, DeJonckheere M, et al. Misconceptions, preferences and barriers to alcohol use disorder treatment in alcohol-related cirrhosis. J Substance Abuse Treat 2018;91:20–27.
[11] Nguyen-Khac E, Thiele M, Voican C, et al. Non-invasive diagnosis of liver fibrosis in patients with alcohol-related liver disease by transient elastography: an individual patient data meta-analysis. Lancet Gastroenterol Hepatol 2018;3:614–625.
[12] Walenbergh SM, Houben T, Hendrikx T, et al. Plasma cathepsin D levels: a novel tool to predict pediatric hepatic inflammation. Am J Gastroenterol 2015;110:462–470.
[13] Walenbergh SM, Houben T, Rensen SS, et al. Plasma cathepsin D correlates with histological classifications of fatty liver disease in adults and responds to intervention. Sci Rep 2016;6:38278.
[14] Ding L, Houben T, Oligschlaeger Y, et al. Plasma cathepsin D activity rather than levels correlates with metabolic parameters of type 2 diabetes in male individuals. Front Endocrinol (Lausanne) 2020;11:575070.
[15] Kharbanda KK, McVicker DL, Zetterman RK, et al. Ethanol consumption reduces the proteolytic capacity and protease activities of hepatic lysosomes. Biochim Biophys Acta 1995;1245:421–429.
[16] Thomes PG, Ehlers RA, Trambly CS, et al. Multilevel regulation of autophagosome content by ethanol oxidation in HepG2 cells. Autophagy 2013;9:63–73.
[17] Thiele M, Madsen BS, Hansen JF, et al. Accuracy of the enhanced liver fibrosis test vs FibroTest, elastography, and indirect markers in detection of advanced fibrosis in patients with alcoholic liver disease. Gastroenterology 2018;154:1369–1379.
[18] Lindvig KP, Hansen TL, Madsen BS, et al. Diagnostic accuracy of routine liver function tests to identify patients with significant and advanced alcohol-related liver fibrosis. Scand J Gastroenterol 2021;56:1088–1095.
[19] **Thiele M**, **Suvitaival T**, **Trošt K**, et al. Sphingolipids are depleted in alcohol-related liver fibrosis. Gastroenterology 2023;164:1248–1260.
[20] Israelsen M, Juel HB, Detlefsen S, et al. Metabolic and genetic risk factors are the strongest predictors of severity of alcohol-related liver fibrosis. Clin Gastroenterol Hepatol 2022;20:1784–1794.e1789.
[21] Trošt K, Ahonen L, Suvitaival T, et al. Describing the fecal metabolome in cryogenically collected samples from healthy participants. Sci Rep 2020;10:885.
[22] Niu L, Thiele M, Geyer PE, et al. Noninvasive proteomic biomarkers for alcohol-related liver disease. Nat Med 2022;28:1277–1287.
[23] Glen J, Floros L, Day C, et al. Non-alcoholic fatty liver disease (NAFLD): summary of NICE guidance. BMJ 2016;354:i4428.
[24] Nobili V, Parkes J, Bottazzo G, et al. Performance of ELF serum markers in predicting fibrosis stage in pediatric non-alcoholic fatty liver disease. Gastroenterology 2009;136:160–167.
[25] Lichtinghagen R, Pietsch D, Bantel H, et al. The Enhanced Liver Fibrosis (ELF) score: normal values, influence factors and proposed cut-off values. J Hepatol 2013;59:236–242.
[26] Fagan KJ, Pretorius CJ, Horsfall LU, et al. ELF score >/=9.8 indicates advanced hepatic fibrosis and is influenced by age, steatosis and histological activity. Liver Int 2015;35:1673–1681.
[27] Dietrich CF, Bamber J, Berzigotti A, et al. EFSUMB guidelines and recommendations on the clinical use of liver ultrasound elastography, update 2017 (short version). Ultraschall Med 2017;38:377–394.
[28] European Association for Study of L. Asociacion Latinoamericana para el Estudio del H. EASL-ALEH Clinical Practice Guidelines: Non-invasive tests for evaluation of liver disease severity and prognosis. J Hepatol 2015;63:237–264.
[29] Thiele M, Madsen BS, Procopet B, et al. Reliability criteria for liver stiffness measurements with real-time 2D shear wave elastography in different clinical scenarios of chronic liver disease. Ultraschall Med 2017;38:648–654.
[30] de Franchis R, Baveno VIF. Expanding consensus in portal hypertension: report of the Baveno VI Consensus Workshop: stratifying risk and individualizing care for portal hypertension. J Hepatol 2015;63:743–752.
[31] Liangpunsakul S, Crabb DW. Early detection of alcoholic liver disease: are we a step closer? Gastroenterology 2016;150:29–31.
[32] Brunt EM, Kleiner DE, Wilson LA, et al. Nonalcoholic fatty liver disease (NAFLD) activity score and the histopathologic diagnosis in NAFLD: distinct clinicopathologic meanings. Hepatology 2011;53:810–820.
[33] Israelsen M, Torp N, Johansen S, et al. Validation of the new nomenclature of steatotic liver disease in patients with a history of excessive alcohol intake: an analysis of data from a prospective cohort study. Lancet Gastroenterol Hepatol 2024;9:218–228.
[34] DeLong ER, DeLong DM, Clarke-Pearson DL. Comparing the areas under two or more correlated receiver operating characteristic curves: a nonparametric approach. Biometrics 1988;44:837–845.
[35] Chao X, Wang S, Hlobik M, et al. Loss of hepatic transcription factor EB attenuates alcohol-associated liver carcinogenesis. Am J Pathol 2022;192:87–103.
[36] **Bala S**, **Babuta M**, Catalano D, et al. Alcohol promotes exosome biogenesis and release via modulating rabs and miR-192 expression in human hepatocytes. Front Cel Dev Biol 2021;9:787356.
[37] **Babuta M**, **Furi I**, **Bala S**, et al. Dysregulated autophagy and lysosome function are linked to exosome production by micro-RNA 155 in alcoholic liver disease. Hepatology 2019;70:2123–2141.
[38] Dagur RS, New-Aaron M, Ganesan M, et al. Alcohol-and-HIV-Induced lysosomal dysfunction regulates extracellular vesicles secretion in vitro and in liver-humanized mice. Biology (Basel) 2021;10.
[39] Paton A. Alcohol in the body. BMJ 2005;330:85–87.
[40] Guilliams M, Scott CL. Liver macrophages in health and disease. Immunity 2022;55:1515–1529.
[41] Malherbe DC, Messaoudi I. Transcriptional and epigenetic regulation of monocyte and macrophage dysfunction by chronic alcohol consumption. Front Immunol 2022;13:911951.

[42] Lowe PP, Morel C, Ambade A, et al. Chronic alcohol-induced neuroinflammation involves CCR2/5-dependent peripheral macrophage infiltration and microglia alterations. J Neuroinflammation 2020;17:296.
[43] Luo J. Autophagy and ethanol neurotoxicity. Autophagy 2014;10:2099–2108.
[44] Lee JH, Yang DS, Goulbourne CN, et al. Faulty autolysosome acidification in Alzheimer's disease mouse models induces autophagic build-up of Abeta in neurons, yielding senile plaques. Nat Neurosci 2022;25:688–701.
[45] Ridler C. Parkinson disease: lysosome storage disorder risk genes linked to PD. Nat Rev Neurol 2018;14:3.
[46] Moreno C, Mueller S, Szabo G. Non-invasive diagnosis and biomarkers in alcohol-related liver disease. J Hepatol 2019;70:273–283.
[47] Deeks JJ, Altman DG. Diagnostic tests 4: likelihood ratios. BMJ 2004;329:168–169.
[48] Livingston M, Callinan S. Underreporting in alcohol surveys: whose drinking is underestimated? J Stud Alcohol Drugs 2015;76:158–164.
[49] Levy RE, Catana AM, Durbin-Johnson B, et al. Ethnic differences in presentation and severity of alcoholic liver disease. Alcohol Clin Exp Res 2015;39:566–574.

Keywords: alcohol use disorder; alcohol-related liver disease; persistent heavy drinking; cathepsin D.
Received 5 December 2023; received in revised form 26 April 2024; accepted 2 May 2024; Available online 9 May 2024

Journal of Hepatology, Volume *6*

Supplemental information

Plasma cathepsin D as an early indicator of alcohol-related liver disease

Mengying Li, Tom Houben, Albert V. Bitorina, Dennis M. Meesters, Mads Israelsen, Maria Kjærgaard, Ger H. Koek, Tim Hendrikx, JefVerbeek, Aleksander Krag, Maja Thiele, and Ronit Shiri-Sverdlov

Plasma cathepsin D as an early indicator of alcohol-related liver disease

Mengying Li, Tom Houben, Albert V. Bitorina, Dennis M. Meesters, Mads Israelsen, Maria Kjærgaard, Ger H. Koek, Tim Hendrikx, Jef Verbeek, Aleksander Krag, Maja Thiele, Ronit Shiri-Sverdlov

Table of contents

Fig. S1……………………………………………………………………………2
Fig. S2……………………………………………………………………………3
Fig. S3……………………………………………………………………………4
Table S1…………………………………………………………………………5
Table S2…………………………………………………………………………7
Table S3…………………………………………………………………………8
Table S4…………………………………………………………………………9
Table S5………………………………………………………………………...10
Table S6………………………………………………………………………...11
Table S7………………………………………………………………………...12
Table S8………………………………………………………………………...13

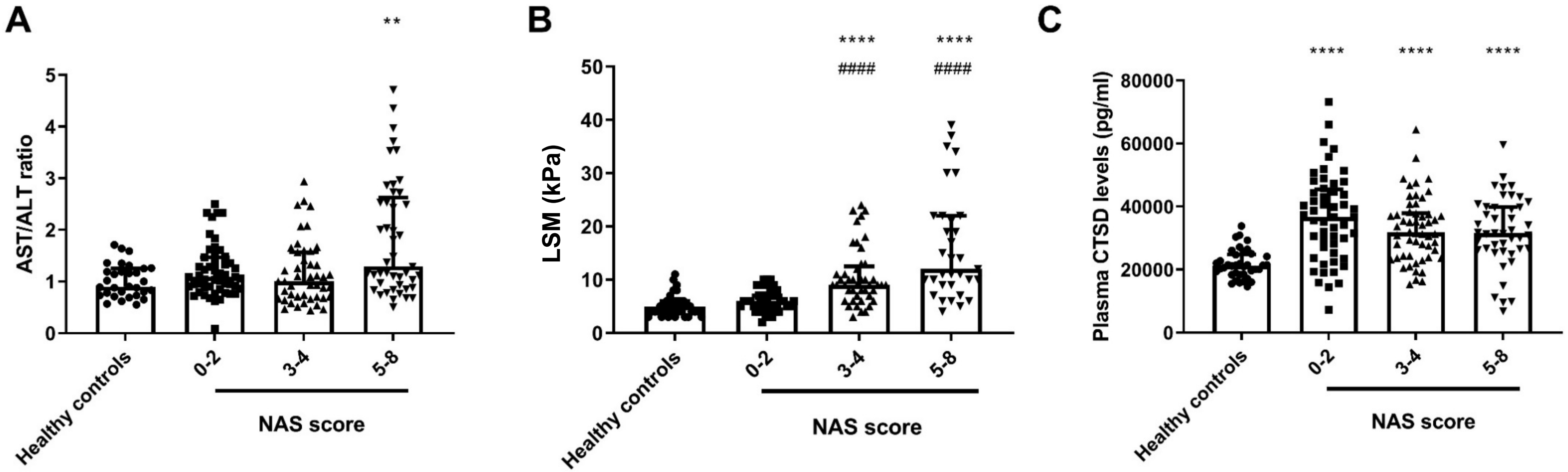

Fig. S1: Plasma CTSD levels and hepatic disease markers increased in MetALD/ALD patients classified according to NAS score

AST/ALT ratio, liver stiffness measurement (LSM) and CTSD levels were analyzed in healthy controls (n=40) and MetALD/ALD patients (n=152) and classified according to the NAFLD NAS-CRN system. The composite NAS score consisted of the sum of steatosis, ballooning and lobular inflammation. ** $p<0.01$, **** $p<0.0001$ compared to healthy controls and $^{\#\#\#\#}$ $p<0.0001$ compared to NAS score 0 (Kruskal-Wallis test). All error bars are median with interquartile range.

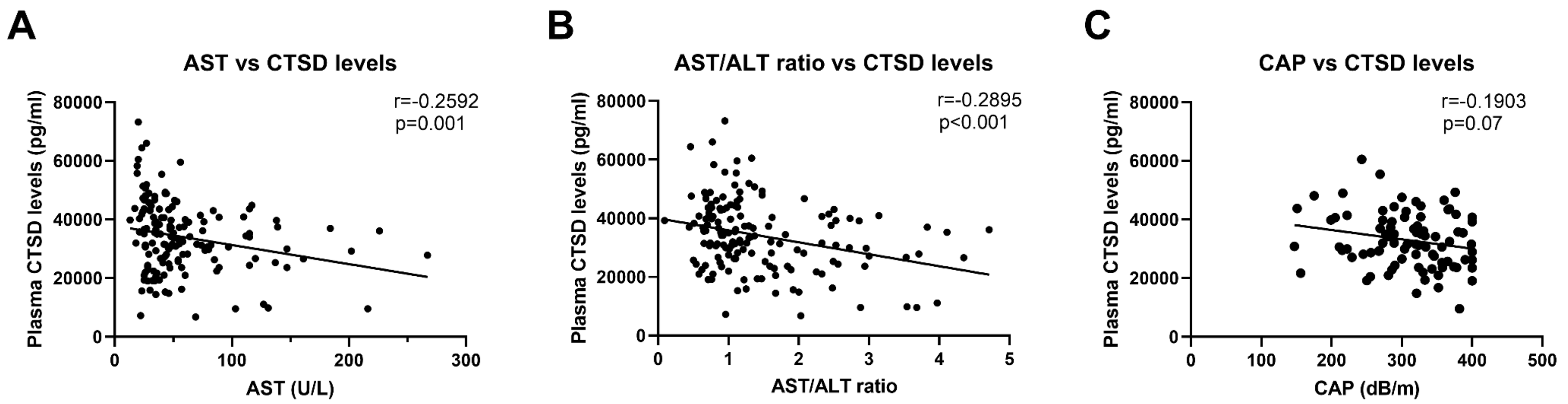

Fig. S2: Plasma CTSD levels correlated with AST and AST/ALT ratio in MetALD/ALD patients.

(A) Plasma CTSD levels vs. AST (n=148), (B) plasma CTSD levels vs. AST/ALT ratio (n=148) and (C) and plasma CTSD levels vs. CAP (n=89). Spearman's correlation was performed. $p<0.05$ was considered statistically significant.

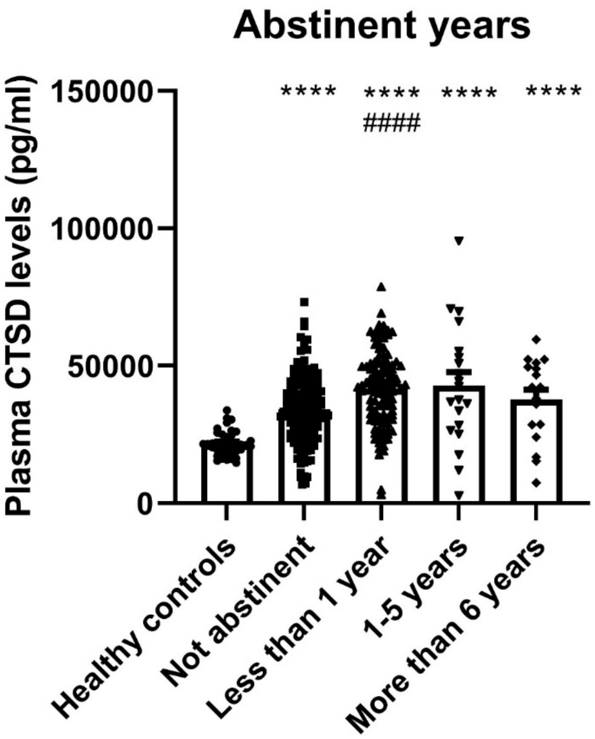

Fig. S3: Plasma CTSD levels during prolonged periods of self-reported abstinence

**** p<0.0001 compared to healthy controls and # # # # p<0.0001 compared to patients that were not abstinent (indicated in graph as ‘not abstinent’) (Kruskal-Wallis test). All error bars are median with interquartile range. (healthy controls, n=38; not abstinent, n=156; less than 1 year of abstinence, n=112; 1-5 years of abstinence, n=20; more than 6 years of abstinence, n=17)

Table S1: Clinical characteristics of MetALD/ALD patients based on NAS score

Parameters	Healthy controls (n=40)	NAS score 0 (n=20)	NAS score 1-4 (n=86)	NAS score 5-8 (n=45)	p-value (score 0 vs. healthy controls)	p-value (score 0 vs. score 1-4)	p-value (score 0 vs. score 5-8)
**Gender (F/M)**	9/31	6/14	19/67	6/39	0.535	0.458	0.165
**Age (y)**	53±1.6	50±2.8	57±1.1	58±1.4	0.243	0.004*	0.004*
**BMI (kg/m2)**	26±0.5	26±1.0	28±0.7	27±0.8	0.546	0.054	0.186
**HOMA-IR**	2.8±0.4	7.2±5.2	5.9±0.9	7.0±1.1	0.406	0.661	0.939
**Insulin (pmol/L)**	69.4±8.0	109.9±59.0	129.7±17.6	134.0±15.4	0.349	0.669	0.599
**Fasting glucose (mmol/L)**	5.9±0.2	6.4±0.4	7.0±0.2	7.4±0.3	0.194	0.228	0.091
**Hemoglobin A1c (mmol/mol)**	37±1.0	38±2.8	37±1.2	37±1.4	0.918	0.977	0.756
**LDL cholesterol (mmol/L)**	3.3±0.1	2.8±0.2	2.8±0.1	2.6±1.1	0.056	0.989	0.393
**HDL cholesterol (mmol/L)**	1.3±0.08	1.4±0.11	1.5±0.06	1.5±0.11	0.796	0.430	0.306
**Triglyceride (mmol/L)**	1.3±0.1	1.8±0.2	2.2±0.2	1.4±0.1	0.043*	0.482	0.098

**CAP (dB/m)**	270±9	227±25	313±8	314±11	0.070	0.001*	0.001*
**ALT (U/L)**	31±3	26±3	49±5	58±4	0.329	0.040*	0.000*
**AST (U/L)**	28±2	27±2	47±3	91±8	0.895	0.000*	0.000*
**AST/ALT ratio**	1.01±0.06	1.16±0.09	1.30±0.09	1.82±0.17	0.148	0.273	0.001*
**LSM (kPa)**	7.0±1.5	5.4±0.5	13.6±1.6	27.5±3.7	0.471	0.000*	0.000*
**MELD**	6.7±0.1	6.6±0.2	7.2±0.2	8.0±0.3	0.498	0.208	0.002*
**CRP (mg/L)**	3.2±0.5	3.6±1.5	7.4±2.7	10.8±2.4	0.744	0.541	0.014*
**Plasma CTSD levels (pg/mL)**	22858.54±1059.15	37737.03±3403.45	33949.17± 1235.81	32189.32± 1663.63	0.000*	0.215	0.104

Data are represented as mean ± SEM. Data are statistically analysed by performing an independent sample t-test using SPSS software, version 27.0 (SPSS, Chicago, IL, USA). BMI: body mass index; HOMA-IR: homeostasis model of insulin resistance; LDL: low-density lipoprotein; HDL: high-density lipoprotein; CAP: controlled attenuation parameter; ALT: alanine transaminase; AST: aspartate transaminase; LSM: liver stiffness measurement; MELD: model of end stage liver disease; CRP: C-reactive protein. * $p<0.05$ is statistically significant.

Table S2: Plasma CTSD levels independently associated with plasma triglycerides in NAS score 0 MetALD/ALD patients and healthy controls

Dependent variable: plasma CTSD levels

	**LDL cholesterol**			**Triglyceride**		
**Models**	**p value**	**Standardized coefficients β**	**Adjusted R square**	**p value**	**Standardized coefficients β**	**Adjusted R square**
**Model 1**	0.242	-0.156	0.007	0.030*	0.286	0.065
**Model 2** Model1+age	0.668	-0.060	0.051	0.034*	0.270	0.123
**Model 3** Model 2+BMI	0.690	-0.057	0.034	0.026*	0.293	0.116
**Model 4** Model 3+sex	0.698	-0.055	0.025	0.034*	0.285	0.103

Data were analyzed by multiple linear regression: Model 1, simple regression; Model 2, model 1 + adjustment for age; Model 3, model 2 + adjustment for BMI; Model 4, model 3 + adjustment for sex. * $p<0.05$ is statistically significant.

Table S3: Plasma CTSD levels are associated with hepatic disease markers in MetALD/ALD patients

Dependent variable: plasma CTSD levels

	LSM			**AST/ALT ratio**			**AST**			**ELF**			**GGT**		
**Models**	**p value**	**Standardized coefficients β**	**Adjusted R square**	**p value**	**Standardized coefficients β**	**Adjusted R square**	**p value**	**Standardized coefficients β**	**Adjusted R square**	**p value**	**Standardized coefficients β**	**Adjusted R square**	**p value**	**Standardized coefficients β**	**Adjuste R squar**
**Model 1**	0.005*	-0.231	0.047	0.000*	-0.306	0.087	0.004*	-0.238	0.050	0.045*	-0.163	0.020	0.052	-0.158	0.025
**Model 2** Model1+age	0.007*	-0.224	0.049	0.000*	-0.302	0.086	0.003*	-0.240	0.052	0.072	-0.151	0.015	0.043*	-0.165	0.034
**Model 3** Model 2+BMI	0.007*	-0.224	0.042	0.000*	-0.347	0.093	0.002*	-0.259	0.050	0.070	-0.154	0.009	0.039*	-0.173	0.035
**Model 4** Model 3+sex	0.009*	-0.218	0.038	0.000*	-0.344	0.092	0.002*	-0.259	0.051	0.098	-0.142	0.008	0.045*	-0.168	0.043

Data were analysed by multiple linear regression: Model 1, simple regression; Model 2, model 1 + adjustment for age; Model 3, model 2 + adjustment for BMI; Model 4, model 3 + adjustment for sex. * $p<0.05$ is statistically significant.

Table S4: Overview of the comparison between the different single AUCs

	Standard Error [a]	z statistic	95% Confidence Interval[b]	P-value
CTSD levels vs. AST/ALT ratio	0.0702	2.689	0.0512 - 0.326	P=0.0072
CTSD levels vs. TE	0.0609	0.664	-0.0789 - 0.160	P=0.5070
AST/ALT ratio vs. TE	0.0728	2.037	0.00561 - 0.291	P=0.0416

[a] DeLong et al., 1988

[b] Binomial exact

Table S5: Different statistical criteria related to the diagnostic value of plasma CTSD, AST/ALT ratio and TE for distinguishing MetALD/ALD from healthy controls

	Cut-off value	Sensitivity %	Specificity %	PPV %	NPV %	LR+	LR-	Accuracy%
CTSD levels	≥21933.10 (pg/ml)	87	68	92	53	2.7	0.2	83
LSM	≥6.5 (kPa)	69	74	92	34	2.7	0.4	70
AST/ALT ratio	≥1.37	36	90	94	24	3.7	0.7	45

PPV: positive predictive value; NPV: negative predictive value; LR+: positive likelihood ratio; LR-: negative likelihood ratio.

Table S6: Overview of the comparison between the different AUCs

	Standard Error [a]	z statistic	95% Confidence Interval[b]	P-value
AST/ALT ratio vs. CTSD + AST/ALT ratio	0.0527	4.609	0.140 - 0.346	P<0.0001
LSM vs. CTSD + LSM	0.0418	2.229	0.0113 - 0.175	P = 0.0258
AST/ALT ratio + LSM vs. CTSD + AST/ALT ratio + LSM	0.0467	3.717	0.0821 - 0.265	P = 0.0002

[a] DeLong et al., 1988

[b] Binomial exact

Table S7: Different statistical criteria related to the diagnostic value of combining hepatic disease markers for distinguishing MetALD/ALD from healthy controls

	Sensitivity %	Specificity %	PPV %	NPV %	LR+	LR-	Accuracy%
CTSD + LSM + AST/ALT ratio	85	84	96	55	5.3	0.2	85
CTSD + LSM	85	84	96	55	5.3	0.2	85
CTSD + AST/ALT ratio	70	94	98	40	10.8	0.3	74
AST/ALT ratio + LSM	47	94	97	28	7.3	0.6	55

PPV: positive predictive value; NPV: negative predictive value; LR+: positive likelihood ratio; LR-: negative likelihood ratio.

Table S8: Plasma CTSD levels during prolonged periods of self-reported abstinence

Years of abstinence	Mean plasma CTSD levels (pg/ml)	N
Healthy controls	21735.5	38
No abstinent	34100.3	156
Less than 1 year	41688.2	112
1-5 years	42786.3	20
6-10 years	30851.4	7
11-20 years	50079.5	6
21-30 years	43824.3	2
more than 30 years	18074.0	2
Total	37654.7	305

Data are represented as mean
